# Signature of an aggregation-prone conformation of tau

**DOI:** 10.1038/srep44739

**Published:** 2017-03-17

**Authors:** Neil A. Eschmann, Elka R. Georgieva, Pritam Ganguly, Peter P. Borbat, Maxime D. Rappaport, Yasar Akdogan, Jack H. Freed, Joan-Emma Shea, Songi Han

**Affiliations:** 1 Department of Chemistry and Biochemistry, University of California Santa Barbara, Santa Barbara, California, 93106, USA; 2 National Biomedical Center for Advanced ESR Technology, Cornell University, Ithaca, New York, 14853, USA; 3 Department of Chemistry and Chemical Biology, Cornell University, Ithaca, New York, 14853, USA

## Abstract

The self-assembly of the microtubule associated tau protein into fibrillar cell inclusions is linked to a number of devastating neurodegenerative disorders collectively known as tauopathies. The mechanism by which tau self-assembles into pathological entities is a matter of much debate, largely due to the lack of direct experimental insights into the earliest stages of aggregation. We present pulsed double electron-electron resonance measurements of two key fibril-forming regions of tau, PHF6 and PHF6*, in transient as aggregation happens. By monitoring the end-to-end distance distribution of these segments as a function of aggregation time, we show that the PHF6^(*)^ regions dramatically extend to distances commensurate with extended β-strand structures within the earliest stages of aggregation, well before fibril formation. Combined with simulations, our experiments show that the extended β-strand conformational state of PHF6^(*)^ is readily populated under aggregating conditions, constituting a defining signature of aggregation-prone tau, and as such, a possible target for therapeutic interventions.

Tau is an intrinsically disordered protein (IDP), whose physiological function appears to be mainly that of stabilizing microtubules, but is found also as a principle pathological component of neurofibrillary tangles (NFT) in the brain^1^,^2^,^3^,^4^. These NFT brain inclusions are the hallmark of a set of neurodegenerative diseases known as Tauopathies, which include Alzheimer’s disease^5^. Within NFTs, tau forms insoluble, high molecular weight, filaments. *In vitro*, tau can be induced to form fibrillar filaments with morphology similar to those found in NFTs^6^,^7^. Recent *in vivo* studies reporting that the fibrillization of tau spreads across cells or tissues have intensified the interest in identifying the seed and mechanism of tau fibrillization, currently unknown^8^,^9^,^10^,^11^,^12^. Missing in any model that tracks tau from a mutation to a distinctive fibril morphology to a pathological pattern of tau inclusions^13^,^14^ is a molecular-level understanding of tau misfolding events that may accompany or precede fibrillization.

Full-length tau, as well as many of their protein and peptide fragment variants are IDPs that are highly soluble and stable *in vitro*, and do not self-assemble under physiological concentrations and solution conditions, even when stored over a prolonged time course. Therefore, the initiation of tau fibrillization *in vitro* typically requires an activator, such as negatively charged macromolecules or surfaces that have been shown to be effective^1^,^15^, including heparin^16^,^17^, lipid membrane surfaces^18^ and RNA^19^. This implies that the conformational ensemble assumed by the solvated IDP, tau, constitutes a stable state. The question is what the defining event is that results in the lowering of the barrier towards fibril formation—our guiding hypothesis is that it is a dramatic shift in the conformational landscape of tau induced by function-altering environmental factors in the earliest stages of aggregation.

Human tau has six known isoforms, of which the longest one is 441 residues long. It contains four microtubule binding (MTB) repeats, R1 to R4 (Fig. 1). Here, we study the truncated variant of this 441 residue isoform, Δtau187 G272C/S285C, which spans residues 255–441 containing all four MTB repeats to the C-terminus (Fig. 1), with the aggregation-impeding N-terminus truncated. Δtau187 forms neat fibrils (see Supplementary Fig. S1) with cross-β sheet structures characteristics of full length tau^20^. The PHF6^*^ (VQIINK) segment as part of the second MTB repeat, R2, and PHF6 (VQIVYK) as part of the third MTB repeat, R3, are the key building blocks that pack to form the β-sheet structured core of mature tau fibrils^16^,^20^,^21^,^22^. Crucially, the PHF6^(*)^ repeat domains of tau stack into highly ordered, neat, β-sheets up the fibril axis, in which these hexapeptide segments adopt fully extended conformations^6^,^23^. Thus, when looking to identify conformational transformation that tau may undergo, we focus on distance changes across sites that are flanking the PHF6^(*)^ region, of stable tau proteins and model peptides in solution state, before and after initiating aggregation. We employ site-directed mutagenesis to cysteine and spin labeling (SDSL) of two sites flanking the PHF6^(*)^ region to perform double electron-electron resonance (DEER) measurements to extract distance and distance distribution as a function of aggregation time. We set out to test a new paradigm for the aggregation of IDPs, namely that distinct conformational ensembles of IDPs represent distinct states, such as the “stable” vs. “aggregation-prone” states of Tau.

## Results

### Heparin extends the PHF6* and PHF6 region of tau

The distances flanking the PHF6* segment of two tau fragments, R2/12 and R2/14, and a tau protein variant, Δtau187, were measured in their stable solution state, as well as upon initiation of aggregation by addition of heparin, as a function of aggregation time. It is worth noting that MTSL spin labeled tau is mixed with MTSL-analogue (non-EPR active) labeled tau at 1:15 down to 1:80 ratios in order to ensure that intra-protein distances are measured (see Supplementary Fig. S2). Ku-band (17.3 GHz) DEER measurements across sites 272 and 285 flanking the PHF6* region of Δtau187 reveal a relatively short average distance of 3.16 nm, before heparin is added to the solution (Fig. 2, panel a). Upon heparin addition, a dramatic shift in the average distance is found towards an extended state that spans 4.35 nm across the spin labels of sites 272 and 285.

Time-resolved DEER measurement of freeze-quenched tau samples at different aggregation times reveals that this extension is essentially complete within ~10 minutes of adding heparin to the tau sample, which is unexpectedly rapid considering the fibril formation kinetics reaches a plateau only after 12–16 hours of aggregation time (see Supplementary Fig. S3). It is important to note that DEER measurements capture the average distribution of all spin labeled molecules of the sample, so that the whole ensemble of conformations is captured with DEER-derived intra-spin distances^24^,^25^. Thus, looking at the results presented in Fig. 2 (panel a), we can conclude that the tau population with conformers displaying, on average, a short G272C-S285C distance of 3.16 nm has quantitatively (91% of Δtau187) transformed into conformers displaying an extended G272C-S285C distance of 4.35 nm, within ~10 minutes of heparin addition. Remarkably, the results in Fig. 2 (panel a) show that a surprisingly large population of approximately 55% of Δtau187 has already undergone this dramatic conformational extension within the dead-time of mixing tau and heparin and loading into the sample tube, referred to as “right after heparin addition” in the figure legend, which is about ~1 minute. Crucially, the remaining 45% of Δtau187 can be represented with the original distance distribution as observed with stable tau, i.e. before heparin addition (see Fig. 2c and Table 1 for population analysis). In other words, the experimental distance distribution can be described with a 2-state model, with one state characterized as compact and the other state as extended with respect to the PHF6* segment. The compact-around-PHF6* ensemble represents stable tau conformers in solution state and the extended-around-PHF6* ensemble represents tau *en route* to aggregation that involve a mixture of solution and intermediate species. Crucially, the majority of tau before and within ~10 minutes of heparin addition are solution state species, and thus the distances refer to intra-protein distances of monomer conformations of tau in different dynamic assembly states, ranging from monomer, dimer to lower order oligomers. We can rule out that a significant population of tau has formed fibrillar species within ~10 minutes of heparin addition, based on β-sheet content formed during this time as determined by quantitative EPR lineshape analysis (using the same total tau concentration of 800 μm as in DEER, presented in Supplementary Fig. S4). We identify that the β-sheet content is clearly seen to be adopted by less than 15% of tau after 2 minutes, less than 20% of tau after 20 min and around 50% within 1 hour of adding heparin, confirming that the conformational changes are initially adopted by non-fibrillar species. Thus, we assign the compact and extended distances around PHF6* to be representative signatures of S and S* states, respectively, in which S or S*refers to solution populations, with further verification to follow. With increasing aggregation time, the population of S* rapidly reaches 91% after 10 minutes and 100% after 1 hour of heparin addition, with no need to invoke the presence of new populations of conformers of tau around the PHF6* region. The S-to-S* transformation is complete, well before fibril formation is complete as detectable by ThT fluorescence kinetics (Supplementary Fig. S3) and β-sheet content by EPR (Supplementary Fig. S4). However, with currently available measurement tools, it is difficult to resolve whether this conformational transformation precedes the formation of small and dynamic oligomers.

**Figure 1:**
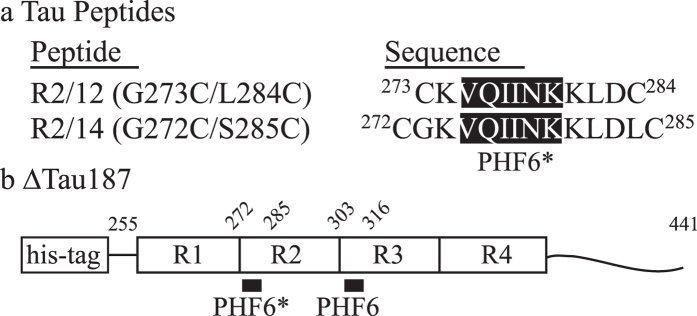
Sequence of tau peptides and schematic of Δtau187. A (**a**) 12-mer peptide R2/12 (G273C/L284C) and a 14-mer peptide R2/14 (G272C/S285C), and (**b**) Δtau187, truncated from the full length, 441 residue tau between residues 255–441. Δtau187 includes all four microtubule binding repeats (MTBRs) to the C-terminus. Two Δtau187 constructs were made with double cysteine mutations in order to measure distances across both PHF6 regions, including Δtau187 G272C/S285C and Δtau187 G303C/S316C.

**Figure 2:**
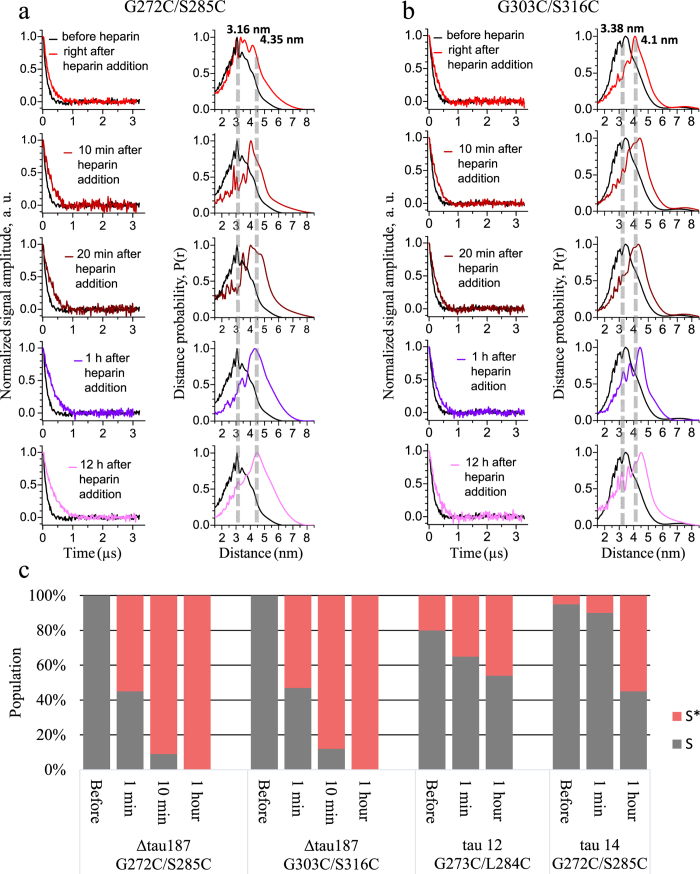
DEER time domain signal and corresponding distance distribution of Δtau187 G272C/S285C and G303C/S316C in addition to time-course of populations S and S* as a function of aggregation time for all double mutants. Background subtracted time-domain DEER signals (**a** and **b**, left panels) and reconstructed distances and distance distributions (**a** and **b**, right panels) of spin-labeled Δtau187 mutants G272C/S285C (panel a) and G303C/S316C (panel b) are shown. In each panel of part a and b, the data obtained without heparin (in black) are overlapped with those after incubation with 200 μM heparin (in shades of red and magenta). The incubation times are indicated in the figure. Visually, the distance maxima shift to higher distances upon addition of heparin. The DEER signals, collected at 17.3 GHz (Ku band), are normalized to be unity at zero time, and the distance distributions plotted normalized to unity at their maxima. The magnetic dilution used was 1:30 spin labeled-to-diamagnetically labeled protein for G303C/S316C samples. Time-course of the compact (S, grey) and extended (S*, red) populations for all double mutants Δtau187 G272C/S285C, Δtau187 G303C/S316C, tau12 G273C/L284C and tau 14 G272C/S285C are shown in c as a function of aggregation time.

Next, we ask the question whether the conformational extension is also seen across the other hexapeptide region, PHF6. Ku-band DEER measurements across sites 303 and 316 flanking the PHF6 hexapeptide region of Δtau187 (see Fig. 1) were repeated following the same experimental protocol as for examining the PHF6* region. Again, the DEER data (Fig. 2 panel b) reveals a short 303-to-316 distance of 3.38 nm in Δtau187, in solution state. Again, the PHF6 region dramatically extends to an average distance of 4.1 nm, within minutes upon heparin addition, well before macroscopic aggregates or any fibrillar species are detectable^26^. The DEER-derived distance distribution is consistent with the posed 2-state model, in which the S state quantitatively transform into S* state populations. Again, right after (~1 min) adding heparin, 53% of the tau has transformed into the S* state and 88% within 10 minutes, while the transformation is complete after 1 hour of adding heparin (see Fig. 2c and Table 1) and remains unaltered through 12 hours upon initiating aggregation.

Finally, DEER measurements of the short, PHF6* containing, R2 peptides were performed across two different lengths of tau peptides, with the DEER time-domain signal and corresponding distance distributions presented in Supplementary Fig. S6 and results summarized in Table 1. Distances across sites 273 and 284 of R2/12 and across adjacent 272 and 285 sites of R2/14 reveal a mean distance of 3.0 nm for R2/12 and 3.6 nm for R2/14, before heparin is added to the solution. Again, we observe a conformational extension across the R2 region to 4.0 nm in R2/12 and to 4.6 nm in R2/14 upon heparin addition, with the longer distance corresponding to the fully extended conformation around R2. Even though we see similar and dramatic extension of the average distance flanking PHF6*, it is clear that the population of PHF6* distances in the R2/12 and R2/14 peptides in solution is already more shifted from the S towards the S* state compared to in Δtau187, before heparin addition. This can be seen in Fig. 2c and Table 1 as the population of the S* state is already 20% for R2/12 and 5% for R2/14, while it was negligible for Δtau187. This is likely because the comparably hydrophobic R2 peptides, albeit dissolved stably in solution, populates dimer or oligomer states to some degree even before heparin addition, as alluded to in previous studies^27^. Gratifyingly, the population still clearly shifts from dominant compact towards dominant extended distances, represented by S and S* conformer populations that are highly similar to that identified for Δtau187. What is different, however, is not only the non-negligible S* population before heparin addition, but also that the S-to-S* conformation is incomplete, involving approximately 50% of the tau population, in contrast to 100% as found with Δtau187 (see Fig. 2c and Table 1). We believe that this is because a fraction of the peptide population participates in imperfect aggregate formation or folding, instead of packing to neat fibrils that necessitates extended PHF6^(*)^ conformations, according to molecular dynamics models. While similar measurements on a peptide spanning the PHF6 region would nicely complement the Δtau187 G303C/S316C results, such peptides are insufficiently soluble in solution, thereby mitigating the possibility of DEER-based distance measurements of these peptides.

What provides strong credibility for the distance extension across PHF6^(*)^ to be a canonical signature of aggregation-prone tau is the consistent results observed with the 187, 14 and 12 amino acid residue-long tau variants upon heparin addition, compared to before the initiation of aggregation, and the observation that these extended distances remain unaltered through the entire fibril formation and maturation process. Strikingly, the extended distance flanking the PHF6 and PHF6* segment, which corresponds to that of a fully extended and consequentially solvent-exposed R2 conformer, appears within minutes of adding heparin and remains unaltered and extended throughout the aggregation process tracked up to 12 hours after heparin addition

### MD simulation shows that PHF6* adopts compact and extended solution populations

**Figure 3:**
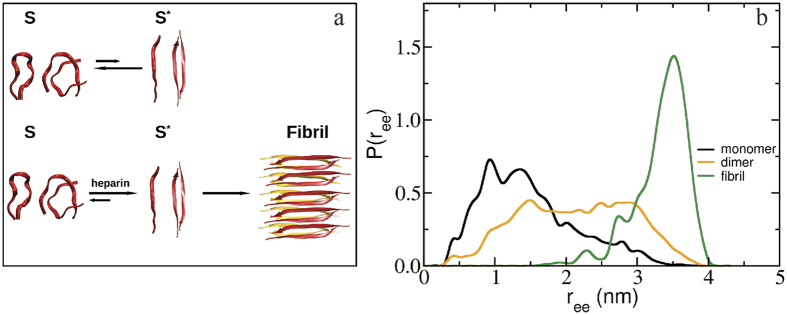
Schematic of the tau-aggregation pathway and simulated end-to-end distances for R2/WT. In the tau-aggregation pathway (**a**), the upper panel depicts the states of Tau prior to adding heparin. Monomer and dimer conformations are shown, in which the individual peptide adopts predominantly a compact, non-aggregating conformation (S) and to a lesser extent, an extended, aggregation-prone conformation (S*). Addition of heparin shifts the equilibrium from S to S*, and leads to fibril formation. Shown are (**b**) end-to-end distance distributions of an individual R2/WT peptide in the context of monomers (black), dimers (orange) and a fibril (green), obtained from MD simulations. The figures include snapshots of the R2/WT monomers and dimers in compact (S state) and extended conformations (S* state), as well as snapshots of a fibril formed by two anti-parallel β-sheets consisting of anti-parallel dimers of R2/WT.

Atomistic *molecular dynamics* (MD) simulations were performed to determine the distance spanning the R2/12 wild type peptide across sites 273 and 284 in the monomer, the dimer (the smallest possible oligomer), as well as in a model fibril state (as described in the Methods section). Figure 3 shows representative snapshots of monomers, dimers and an equilibrated small fibril, the probability distribution of the average 273-to-284 distance of the individual peptides within the monomers, dimer, and fibrillar species. Our earlier simulations of an isolated R2/12 monomer^28^ showed that this peptide is intrinsically disordered, and co-exists between structures with different degrees of compaction, with a preference for compact conformations. In particular, the most populated structure is a hairpin in which the 273-to-284 distance would be on average 0.84 nm. Other compact conformations stabilized by salt-bridges co-exist with the hydrogen-bond stabilized hairpin structures. Extended monomeric conformations are also observed in simulation, but they are rare, as such structures are unstable due to lack of favorable interactions such as hydrogen bonds and salt-bridges. Dimerization leads to an extension of the individual chains, with compact (~1.5 nm) and more extended (~3 nm) conformations coexisting. Once the peptides—in monomer, dimer or larger soluble oligomer forms—become incorporated in a fibril, the 273-to-284 distance distribution shifts distinctly towards even more extended distances averaging around 3.51 nm, corresponding to a significant extension of the individual chain. The largest distance observed in simulations is ~4.0 nm, which corresponds to a fully stretched conformation. Recall, that these fully extended distances up to ~4 nm are in fact dominantly present in the experimental distance distribution data of tau upon initiation of aggregation (see Fig. 2). As can be seen from the snapshot of fibril shown in Fig. 3, most of the chains in the fibril are extended, but do not adopt perfect β-strand structure. This is a result of the small size of the fibril that we model, coupled with thermal fluctuations and dynamics inherent to the simulations. Thus, the experimental mean 273-to-284 distances within the larger fibrils seen experimentally would be larger than the ~3.51 nm we see in simulation.

Importantly, the results from the MD simulation of 273-to-284 distances inform that the DEER-derived extended distances populated within minutes of heparin addition are that of tau conformers that fit to stack into β-sheets, while the distance distribution of stable tau solution is represented by a collection of monomer and dimer populations, as captured by the more compact structures seen by MD simulation (in the absence of heparin). In other words, the initiation of aggregation is marked by a dramatic population shift towards the extended PHF6^(*)^ conformations that are characteristic of fibrils, except that this occurs within the earliest stages of aggregation with >90% of the tau population adopting the extended conformations in solution state. Taken together, the experimentally identified S state population (Table 1) encompasses solution tau species represented by monomer and dimer conformations (see Fig. 3), and possibly include minority populations of lower order oligomers. The S* state (see Table 1), populated upon heparin addition, encompasses solution species of monomers, dimers and soluble oligomers that adopt extended PHF6^(*)^ conformations as found in β-sheet fibrils of tau. We conclude that the inducing of aggregation, in this case by the addition of the fibril-inducing cofactor heparin, is marked by a dramatic shift in population from S towards S* conformers, *en route* to fibrils, well before β-sheet signatures or fibrils are experimentally detectable.

MD simulations further give insight into the arrangement of β-sheets within the fibril of the R2 segment studied. The simulations show that the most stable fibril consists of an antiparallel arrangement of strands within a single β-sheet, with β-sheets further stacking onto other β-sheets in an antiparallel manner. A depiction of the fibril with local distances can be seen in Supplementary Fig. S8. The parallel stacking of sheets led to a slightly less stable fibril, with a slightly smaller average 273-to-284 distance of 3.24 nm (shown in Supplementary Fig. S9). However, parallel arrangements of R2 peptides within a given sheet were not stable (a more detailed discussion is provided in the SI). It should be noted that the specific β-sheet packing of the R2 segment in the stable fibrils built from peptide fragments can be different from that of longer tau protein, as seen with stable antiparallel fibrils made of Aβ16-22 and parallel fibrils made of Aβ9-40^29^,^30^,^31^,^32^. Important for this discussion is that the R2 segment is extended within the β-sheet, regardless of its specific packing.

## Discussion

Changes in the conformation of tau have long been speculated to be a trigger of aggregation, however no specific signature conformations were proposed. As early as 1999, it was suggested that heparin may be capable of extending tau, based on the observation that additional phosphorylation sites become available after the addition of heparin^33^. Since then, some groups have proposed further evidence supporting the hypothesis that heparin is capable of altering the conformational state of tau, but there is no direct experimental report to date on the changes of tau conformations *prior* to fibrillization^34^,^35^,^36^,^37^. Interestingly, a recent MD simulation study showed that Aβ, thought to be a trigger in itself of tau aggregation, can extend the nucleating core of tau which, in turn destabilizes the intra-molecular interactions and allows fibrillization to occur^38^. The emerging concept is that the relatively compact conformation found around the hexapeptide regions of tau in solution state may play a role in hindering tau fibrillization, while there has been a previous report on persistent local structures of type I β-turns near the same hexapeptide region of tau protein in solution, whose role in preventing pathological fibrillization has been speculated^39^.

DEER measurements of intra-protein distance distributions revealed that both the PHF and PHF6* hexapeptide regions of tau assume a comparably compact conformation in stable solution state that completely transforms into a fully extended conformation found in β-sheet fibrils, within minutes of initiating aggregation by the addition of heparin, i.e. well before any fibrillar species or aggregates are detectable. This extended conformation around the PHF6^(*)^ region is maintained throughout 12 hours of aggregation, which is consistent with the finding that the extended conformation is the stable one represented in β-sheet structured fibrillar assembly of tau according to computational models. The DEER-derived distance distribution of all four tau variants (Tau 12 G273C/L284C, Tau 14 G272C/S285C, Tau 187 G272C/S285C, Tau 187 G303C/S316C) studied here can be described with a 2-state model for the ensemble of conformations around PHF6^(*)^—compact and extended—without needing to invoke intermediate or additional conformational species. We designate the conformer population with the compact PHF6^(*)^ as the stable S state and the conformer population with the extended PHF6^(*)^ as the aggregation-prone S* state. The S state still contains many conformations around PHF6^(*)^, but the point is that its ensemble reproducibly represents stable tau. The S* state is characterized by the hydrophobic PHF6^(*)^ segment exposed to solvent and displaying more favorable interaction with another tau peptide than with the solvent, and hence be driven towards self-assembly and subsequent β-sheet formation. We will refer to the S-to-S* transition as misfolding. While this terminology is used in the literature to refer to the protein conformation in the fibrils^40^, *there is no direct experimental report on the misfolding of tau in solution state, preceding fibril formation*. We posit that the S-to-S* misfolding step is rate-limiting in stable, non-pathological, tau proteins. Thus, an initiator is needed that may be changes in external environmental factors in solution—pH, salt, solutes or other cofactors—leading to the population of the S* state^27^,^41^. Additionally, potentially altering the conformer landscape are genetic factors, including tens of different tau missense mutations or exon 10 splice junction mutations that are known to cause a variety of tauopathy phenotypes^13^,^42^, and/or hundreds of combinations of post-translational modifications^43^,^44^. Whatever mechanism induces this misfolding step, it is followed by a cascade of aggregation events leading to the fibrillization of S* species. A recent report shows that dynamic aggregation intermediates that form within minutes of initiating aggregation constitute >40–70% of the total tau population^26^, while this study finds the S* conformers to constitute >50% of the total tau population, again within minutes of initiating aggregation. This shows that we cannot definitely differentiate whether the S-to-S* conformational changes precede the formation of soluble aggregation intermediates or *vice versa*— to do so require a fast-freeze method to quantitatively analyze the S-to-S* transformation kinetics which is outside the scope of this paper, and is thus left for future studies. We can say with certainty, however, that the S-to-S* conformational changes and the early aggregation intermediate formations are major events preceding fibril formation and are *on the pathway* towards fibril formation.

The very concept of a distinct conformational landscape of an intrinsically disordered protein representing distinct states of a protein, such as the “stable” state vs. “aggregation-prone” states of Tau as identified in this study, is a new paradigm for the folding and aggregation of IDP. This finding in fact clarifies key points about the aggregation mechanism of tau. According to the prominent *nucleation polymerization* model^45^,^46^, rare events of fibril formation after 1–2 hours of aggregation autocatalytically seed and accelerate fibril formation. This picture often relies on conventional methods for fibril detection, such as transmission electron microscopy (TEM), fluorescence spectroscopy after ThT(S) staining or light scattering at 350 nm (turbidimetry) that detect fibrils exceeding a critical size and/or concentration threshold, but are insensitive to the appearance of dynamic and small protein assemblies, and are certainly blind to protein conformational changes. Rather, our finding that early oligomer formation and conformational transformation of solution tau involve the majority protein population in the earliest stages of aggregation is consistent with the *nucleation conformational transformation* model^3^,^40^, in which a conformational transformation is needed to overcome the energetically costly stacking of proteins to form fibrils. This latter model is also consistent with a recent literature report that the elongation of fibrils proceeds predominantly by block-wise addition of β-sheet species, not by monomer addition^26^. A critical remaining question is then what is the responsible molecular mechanism and co-factor for stabilizing the S vs the S* state, and whether the S* species are seed-competent to replenish S* from S species, or are simply aggregation-prone. Unraveling the details of the aggregation mechanism is highly relevant for developing adequate therapeutic targets. If fibrillar species are not autocatalytic, strategies to eliminate these species will not prevent aggregation, however if their elongation is energetically favorable, strategies to eliminate these species would still slow fibrillization. If the S to S* conformational transition facilitates aggregation *on pathway* towards fibrillization, strategies to prevent this transition will be ultimately most effective to prevent fibrillization downstream.

## Materials and Methods

### Expression and Purification of Δtau 187

The gene of Δtau 187 which consists of residues 255–441 from full length tau was cloned into the pET28a vector between Nde1 and Xho1 restriction sites with simultaneous mutations at G272C and S285C to make Δtau187 G272C/S285C and simultaneous mutations at G303C and S316C to make Δtau187 G303C/S316C. All mutations were made using site-directed mutagenesis. Each Δtau187 vector was transformed into *E. coli* BL21 (DE3) and grown in 10 mL of LB broth (Fisher Scientific) at 37 °C with shaking. This culture was then used to inoculate a fresh 1 L batch of LB and was incubated at 37 °C with shaking until it reached an optical density of 0.6–0.8 at A_600_. Expression of Δtau 187 was then induced by adding 1 mM final isopropyl β-D-thiogalactopyranoside (IPTG, Fisher Scientific) and incubated for 3 hours. The bacteria was harvested by centrifugation at 4,500 × *g* (Beckman J-10) for 20 min.

Pellets were dissolved in lysis buffer (Tris-HCl, pH = 7.4, 100 mM NaCl, 0.5 mM DTT, 0.1 mM EDTA) supplemented with 1 Pierce protease inhibitor tablet (Thermo Scientific) and PMSF (1 mM). Enzymatic lysis was initiated by the addition of lysozyme (2 mg mL^−1^), DNase (20 μg mL^−1^), and MgCl_2_ (10 mM), and incubated on ice for 30 min. Samples were then sonicated for 20 min using a bath sonicator (Laboratory Supplies Co.). Cell debris was removed by centrifugation at 10,000 rpm at 4 °C for 10 min. Again, PMSF (1 mM) was added and the lysate heated to 65 °C for 12 min. The samples were allowed to cool on ice for 20 min and then centrifuged at 10,000 rpm for 10 minutes to remove the precipitant. The resulting supernatent was loaded onto a Ni-NTA agarose column equilibrated in wash buffer A (20 mM sodium phosphate, pH = 7.0, 500 mM NaCl, 10 mM imidazole, 100 μM EDTA). The column was washed with 20 mL of buffer A, 15 mL buffer B (20 mM sodium phosphate, pH = 7.0, 1 M NaCl, 20 mM imidazole, 0.5 mM DTT, 100 μM EDTA), and again with 10 mL of buffer A. Purified protein was eluted off the column in fractions with buffer C (20 mM sodium phosphate, pH = 7.0, 0.5 mM DTT, 100 mM NaCl), supplemented with varying amounts of imidazole increasing from 100 mM to 300 mM. Fractions were analyzed by SDS-Page and the ones containing pure tau pooled together. The protein was reduced with 5 mM DTT and precipitated by adding equal volumes of methanol and incubating at −20 °C overnight. The resulting precipitated protein was collected by centrifugation at 5,000 rpm at 4 °C for 45 min.

### Spin Labeling of Δtau 187

Purified tau was dissolved in 6 M guanidinium hydrochloride and labeled by adding a 20 fold molar excess of spin label (1-oxyl-2,2,5,5,-tetramethylpyrroline-3-methyl) methanethiosulfonate (MTSL, Toronto Research Chemicals) or the diamagnetic analogue of MTSL (1-Acetoxy-2,2,5,5,-tetramethyl-δ-3-pyrroline-3-methyl) methanethiosulfonate (Toronto Research Chemicals). Excess label was removed by running the labeled protein through a PD-10 desalting column (GE Healthcare) equilibrated in deuterated working buffer (20 mM ammonium acetate, pH = 7.0, 100 mM NaCl, 0.1 mM EDTA). The protein was concentrated using a 3 kDa cutoff centrifugal filter (Amicon). The final protein concentration was determined via absorbance at A_274_ using an extinction coefficient of 2.8 cm^−1^ mM^−1^. Spin labeling efficiency for both Δtau187 mutants was estimated to be near completion.

### Spin Labeling of Tau Peptides

Tau peptides purchased from Genscript were dissolved in 6 M guanidinium hydrochloride along with 10 mM DTT to remove any disulfide bonds. DTT was removed using a 1 kDa dialysis tube (GE Healthcare) and labeled by adding a 20 fold molar excess of spin label MTSL or the diamagnetic analogue of MTSL. The labeling process was carried on overnight at 4 °C. Excess label was removed by running the labeled protein through a PD MidiTrap G-10 desalting column (GE Healthcare) equilibrated with pure water. The spin labeled peptide was then lyophilized and stored at −20 °C. When ready to use, the peptide was dissolved to the desired concentration in working buffer (20 mM ammonium acetate, pH = 7.0). Spin labeling efficiency for both peptides was found to be near 50–60%.

### Transmission Electron Microscopy (TEM)

TEM images were obtained using a JEOL-1230 microscope with an attached ORCA camera paired with an AMT Image Capture Software Version 5.24 (Advanced Microscopy Techniques). Aggregated tau samples contained 100 μM peptide/protein and 25 μM 11 kDa average poly-disperse heparin (Sigma Aldrich) in their respective buffers and were allowed to incubate for 24 hours. It is worth noting that poly-disperse heparin is used due to the lack of availability for mono-disperse heparin. Samples were fixed for 5 min in 1.6% glutaraldehyde (Electron Microscopy Sciences). After fixation, samples were placed on TEM grids (300 mesh, formvar/copper, Electron Microscopy Sciences) for 1 min, rinsed with deionized water, and then stained for 1 min with 2% uranyl acetate.

### Double Electron Electron Resonance (DEER) at Ku-band

Δtau 187 G272C/S285C DEER samples were prepared by mixing respective stock solutions to give 50 μM MTSL labeled Δtau 187 G272C/S285C and 750 μM diamagnetic analogue labeled Δtau 187 G272C/S285C, i.e. a 1:15 ratio needed to suppress intermolecular contributions to the distance distributions. In heparin containing samples, the final concentration of heparin was 200 μM. Δtau 187 G303C/S316C samples differ in using 20 μM MTSL labeled Δtau 187 G303C/S316C and 600 μM analogue labeled Δtau 187 G303C/S316C, i.e. a 1:30 ratio; and heparin when used was in the final concentration of 155 μM. All protein samples contained 30 wt% of sucrose for cryoprotection. Tau peptide samples (G273C/L284C and G272C/S285C) had 25 μM MTSL labeled peptide and 500 μM cysteine-less peptide (i.e. a 20:1 ratio) along with 20% glycerol-*d*_8_. In heparin containing peptide samples, the heparin concentration was 131 μM. D_2_O containing working buffers were used in all cases. The solutions were placed into 2.55 m o.d. × 1.8 mm i.d. pyrex capillary tubes and flash frozen in liquid nitrogen for DEER measurements at required time points after the addition of heparin.

The four-pulse DEER experiments were conducted at 60 K as previously described^24^ using a home-built Ku-band pulse ESR spectrometer^47^ operating at 17.3 GHz. The detection π/2-π-π sequence pulse widths were 16 ns, 32 ns, and 32 ns, respectively. The pump π-pulse was 32 ns, except as noted. The separation between the detection and pump pulse frequencies was 70 MHz. The dipolar evolution times were 3–3.2 μs but as short as ~2 μs for R2 peptides in the samples with heparin. The raw DEER data were processed as described in Georgieva *et al*.^48^. The backgrounds were subtracted out (Supplementary Fig. S10) and inter-spin distances reconstructed by using model-free L-curve Tikhonov regularization^49^ followed by the method of maximum entropy^50^. The experimental distance distributions were fitted using the constrained nonlinear least square curve fitting algorithm built in Origin software (OriginLab) to either a one- or two-Gaussians model yielding the average distances and populations of S and S* states:


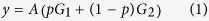



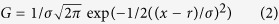


Where *A* is a scaling factor, *p* is the population of S state and the population of S* state is (1 − *p*). For the cases when either S or S* state are not populated, *p* or (1 − *p*) are zeros, respectively. *σ* is the standard deviation, and *r* is the Gaussian mean; *σ* and *r* are in nanometers. The quality of fit was estimated by the R^2^ values (as determined in Origin software), which varied between 0.98 and 0.76 with smaller values in the case of distances for the tau peptides.

### MD Simulations

All-atomistic simulations were performed using GROMACS package (version 4.5.5 and 5.0)^51^. We have used OPLS-AA parameters^51^,^52^,^53^,^54^ for R2/wt peptide (273GKVQIINKKLDL284) and rigid TIP3P model for water^55^. The peptide chains were simulated at their natural charged state (+2 for each chain) and the total charge of the system was neutralized by adding chloride ions. Cut-off for all the non-bonded interactions was set at 1.2 nm. Long-range electrostatic interactions were calculated using particle mesh Ewald method^56^ with a grid-spacing of 0.12 nm. All the bonds of the peptide chains were constrained using LINCS algorithm^57^ and SETTLE algorithm^58^ was used to keep the water molecules fully rigid. The Newtonian equations of motion were solved by a leap-frog integrator^59^ with a time-step of 2 fs. In order to build the R2/wt peptide fibrils we started with an anti-parallel dimer of R2/wt peptide chains, obtained from our previous replica-exchange molecular dynamics simulations (REMD)^60^,^61^,^62^, where the most probable stretched conformation of the dimer was used^63^. Then we built a beta-sheet like structure with five dimers of R2/wt and continued to build the fibril by placing two beta-sheets together in an anti-parallel arrangement (see Figure-Scheme). The fibril containing 20 chains of R2/wt was then solvated in ~10000 water molecules in a cubic box with ~7.0 nm linear dimension. The systems were simulated for 100 ns preceded by 2 ns of equilibration, and the data were analyzed for the first 10–20 ns of the simulations. We performed NpT simulations at 300 K temperature and 1 bar pressure using Nosé-Hoover thermostat^64^,^65^ and Parrinello-Rahman barostat^66^ respectively.

## Additional Information

**How to cite this article:** Eschmann, N. A. *et al*. Signature of an aggregation-prone conformation of tau. *Sci. Rep.* **7**, 44739; doi: 10.1038/srep44739 (2017).

**Publisher's note:** Springer Nature remains neutral with regard to jurisdictional claims in published maps and institutional affiliations.

## Supplementary Material

Supplementary Information

## Figures and Tables

**Table 1:** Population analysis of DEER-derived distance distributions for all tau constructs studied.

Sample	State	Δtau187 G272C/S285C			Δtau187 G303C/S316C			R2/12 G273C/L284C			R2/14 G272C/S285C		
		r	σ	p	r	σ	p	r	σ	p	r	σ	P
before heparin	S	3.16	0.98	1	3.38	0.8	1	3.0	0.56	0.8	3.6	0.75	0.95
	S*	—	—	0	—	—	0	3.75	0.48	0.2	4.35	0.3	0.05
right after heparin	S	3.16	0.98	0.45	3.38	0.86	0.47	3.0	0.62	0.65	3.4	0.75	0.9
	S*	4.1	1.1	0.55	4.4	0.8	0.53	4.0	0.65	0.35	4.6	0.6	0.1
10 min heparin	S	3.16	0.98	0.09	3.38	0.86	0.12	—	—	—	—	—	—
	S*	4.1	1.1	0.91	4.22	0.86	0.88	—	—	—	—	—	—
1 h heparin	S	—	—	0	—	—	0	3.0	0.5	0.54	3.4	0.75	0.45
	S*	4.35	1.1	1	4.1	0.98	1	4.0	0.6	0.46	4.6	0.7	0.55

Experimental distance distributions were fitted to sums of two Gaussians, representing the contributions from two states corresponding to short-distance (S) and long-distance (S*) components. From the Gaussian fittings, the means (*r*), standard deviations (σ) and populations (*P*) of S and S* states were extracted. The average distances and distribution widths, i.e. *r* and σ, are in nanometers. The estimated from Gaussians fitting standard error of the mean for Δtau187 mutants was ±0.06, but for Δtau187 G272C/S285C mutant right after heparin and for tau peptides this value was greater of up to ±0.5 (Supplementary Fig. S6). The fittings were conducted based on S → S* transition model for the tau population.
