# Supplementary material for: Signature of an aggregation-prone conformation of tau
Source: Sci Rep. 2017 Mar 17;7:44739. doi: 10.1038/srep44739 (PMC5356194; doi:10.1038/srep44739)
Supplement: Supplementary Information [file srep44739-s1.pdf]

Signature of an aggregation-prone conformation of tau

Neil A. Eschmann[1], Elka R. Georgieva[2,3,a], Pritam Ganguly[1], Peter P. Borbat[2,3], Maxime D. Rappaport[1,b], Yasar Akdogan[1,c], Jack H. Freed[2,3], Joan-Emma Shea[1], Songi Han[1,*]

[1] Department of Chemistry and Biochemistry, University of California Santa Barbara, Santa Barbara, California, 93106, U.S.A.

[2] National Biomedical Center for Advanced ESR Technology, Cornell University, Ithaca, New York, 14853, U.S.A.

[3] Department of Chemistry and Chemical Biology, Cornell University, Ithaca, New York, 14853, U.S.A.

[*]Corresponding Author: Songi@chem.ucsb.edu

Supplementary Information

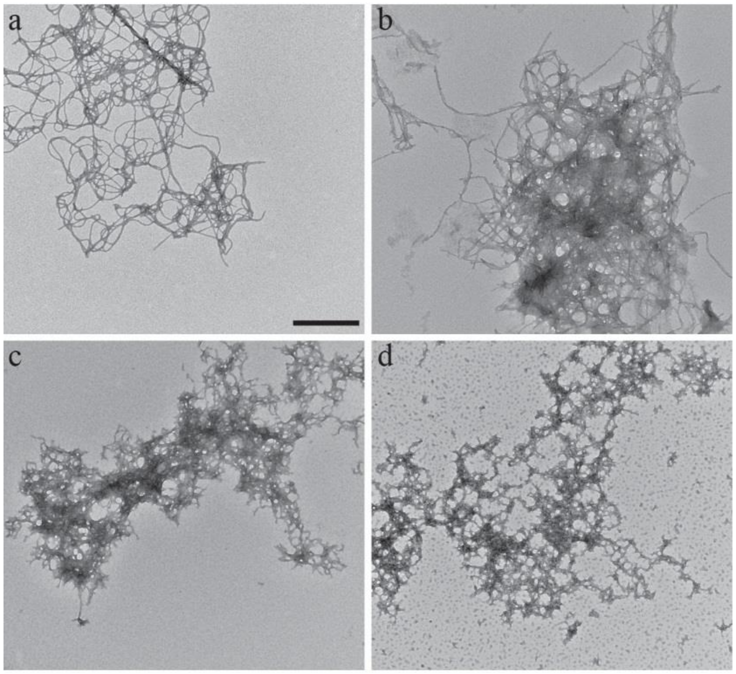

Supplementary Figure S1. TEM images of tau peptides and proteins showing all mutants make fibrillar aggregates including R2/12 G273C/L284C (a), R2/14 G272C/S285C (b), Δtau187 G272C/S285C (c), and Δtau187 G303C/S316C (d). Scale bar = 500 nm.

It was found that each of the tau peptides and proteins used in this study form fibrillar aggregates in the presence of heparin when aggregated by the addition of heparin at a 4:1 ratio of tau to heparin, which is the typical condition used to induce tau aggregation[1]. Both tau peptides R2/12 G273C/L284C and R2/14 G272C/S285C with the nitroxide spin probe MTSL attached form fibrillar aggregates as shown by TEM in Fig. S1 (a) and (b), respectively. Also, the two Δtau187 mutants used in this study, G272C/S285C and G303C/S316C, labeled with MTSL are shown to aggregate as shown in Fig. S1 (c) and (d), respectively. It is worth noting that TEM images generated with these two mutants do not always generate neat fibrils but at times clumpy fibrillar aggregates showing a certain level of disorder. However, ThT staining assays (Fig. S3) in addition to CW EPR lineshape analysis (Fig. S4) verify that the majority of tau forms β-sheet ordered structures. Therefore, the variants studied here are considered valid model systems of the tau protein.

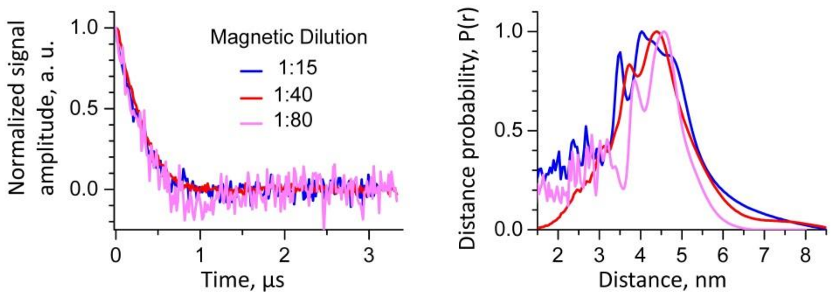

Supplementary Figure S2. DEER data (17.3 GHz) and reconstructed distances for various magnetic dilutions. The data were fitted using L-curve Tikhonov regularization followed by the method of maximum entropy for the cases of 1:15 (blue), 1:40 (red), and 1:80 (magenta) magnetic dilution of MTSL spin labeled by diamagnetically labeled G272C/S285C variants of Δtau187. These produced similar results, differing only in the extent of noise. Each sample was flash frozen 20 minutes after adding the required amount of 11 kDa heparin for a 4:1 ratio of tau:heparin. Samples with a magnetic dilution of 1:15 were made using 50 μM MTSL-labeled and 750 μM analogue labeled proteins, 1:40 samples were made using 25 μM MTSL and 1 mM analogue labeled proteins, and 1:80 samples were made using 5 μM MTSL and 400 μM analogue labeled proteins.

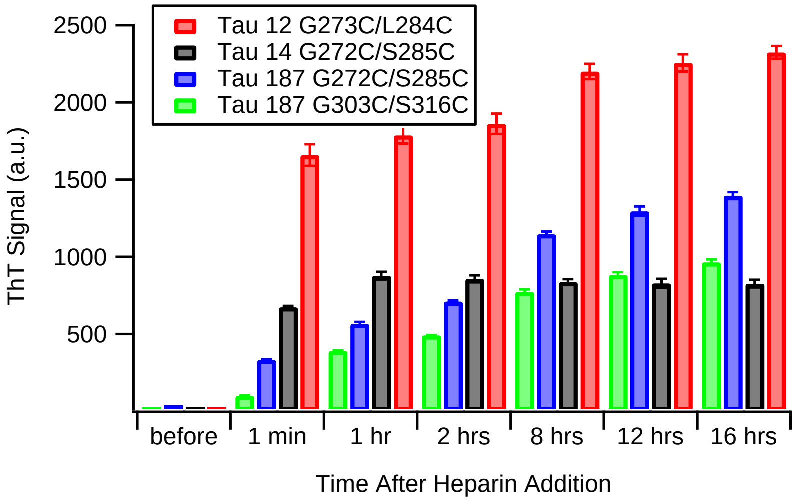

Supplementary Figure S3. Thioflavin T (ThT) kinetic measurements for all Tau mutants. The data show that all mutants are ThT active after incubation with heparin, implying that they all generate β-sheet structured amyloid aggregates including Tau 12 G273C/L284C (red), Tau 14 G272C/S285C (black), Tau 187 G272C/S285C (blue), and Tau 187 G303C/S316C (green). All

samples were run using 25 μM protein, 6.7 μM ThT, and 6.25 μM heparin, and kept at ambient room temperature for the duration of the experiment. Data shown are averaged from three separate experiments and standard deviation shown via error bar.

Fibril formation, as measured by fluorescence spectroscopy of ThT-active fibrils, shows an increase in ThT activity for all mutants over a 12-16 hour period after aggregation is induced by the addition of heparin (see Fig. S3). This provides further evidence that all tau variants used in this study do successfully aggregate and form amyloid fibrils. The trends of ThT activity as a function of aggregation time also show that the formation and maturation of ThT-active β-sheet fibrils takes several hours from Δtau187 proteins, but occurs more rapidly with the 12- or 14-mer tau peptides. However, ThT experiments are conducted at lower tau concentrations compared to the DEER experiment. Therefore, CW-EPR combined with line-shape analysis was completed on Δtau187 labeled with MTSL at site 322 (Fig. S4). It has been previously shown that parallel β-sheets form a distinct single line component in the EPR spectra[2-4] for which the population can be extracted using EPR simulation software such as the MultiComponent analysis software. Based on the population analysis, β-sheet formation steadily increases through 5 hours of adding heparin with ~50% of the Tau population constituting β-sheets and continues to slowly increase to ~60% 10 hours after adding heparin. This time course is in stark contrast to that of the conformational extension around PHF6$^{(*)}$, which is complete within 1 hour of adding heparin.

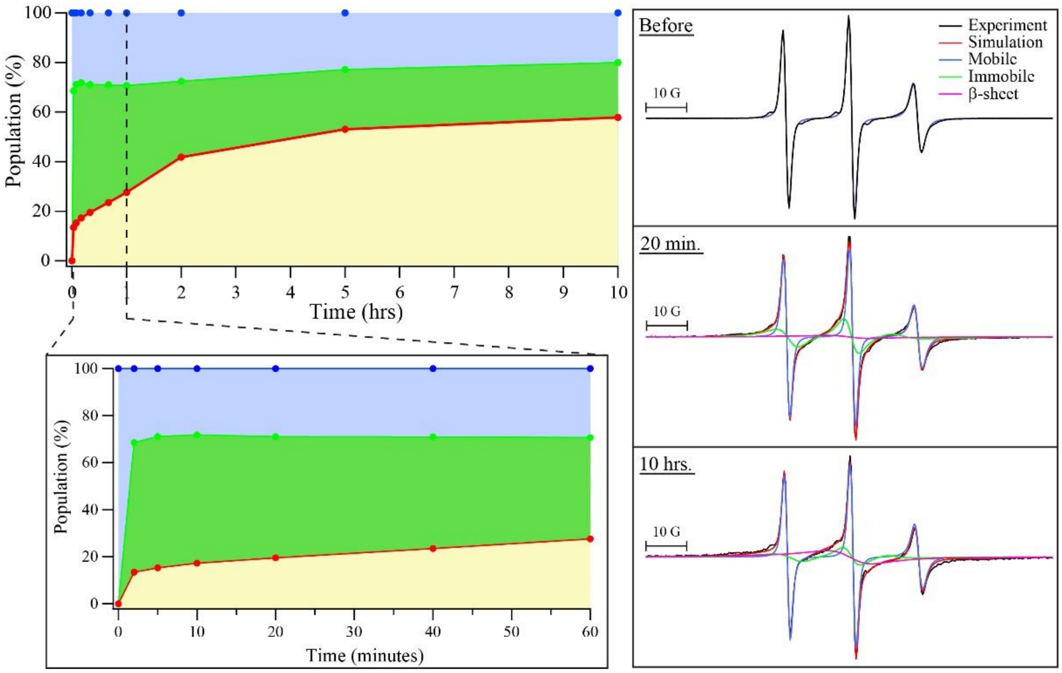

Supplementary Figure S4. Time course for the population of mobile, immobile and β-sheet species of Δtau187 322C upon heparin addition based on CW-EPR line-shape analysis as previously presented[2]. CW-EPR spectra were recorded for 800 μM MTSL-labeled (at site 322) Δtau187 as aggregation progresses after the addition of 200 μM 11 kDa heparin (left panels). Spectra were simulated using the MultiComponent analysis software and mobile (blue), immobile (green) and β-sheet (yellow) components. The full kinetic time-course is shown on top while the bottom shows a zoomed-in region of the first 1 hour of aggregation. Examples of the EPR simulation are shown (right panel) for before, 20 minutes, and 10 hours after heparin addition. Experimental EPR data is shown in black while the simulation is shown in red. Individual components are also displayed including the mobile component (blue), immobile component (green) and β-sheet component (magenta).

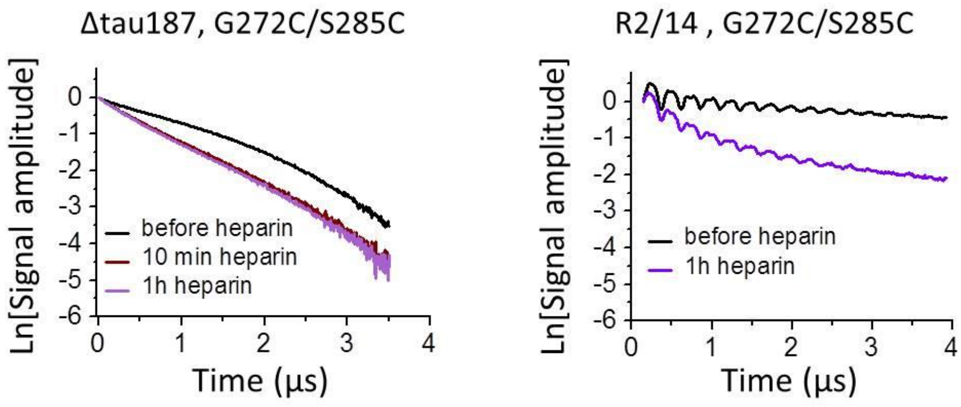

Supplementary Figure S5. The primary echo decays for spin-labeled Δtau187 mutant G272C/S285C (left) and R2/14 peptide (right) on a semi-log scale; these data for R2/12 are similar to those for R2/14, and thus are not shown. The sample conditions (without and with heparin) are indicated in the legends. In the case of R2/14 peptide, D_2O buffer was used, producing visible deuterium ESEEM oscillations in the primary echo decay signal, where a slight decrease in the modulation is noticed upon heparin addition. For both samples, Δtau187 and R2/14, heparin addition shortens the phase memory time (T_m), as judged by the primary echo decay.

The primary echo decays clearly demonstrate significant shortening of the T_m of tau samples that contain heparin, as compared to tau samples without heparin as seen in Fig. S5. This indicates that in all cases the presence of heparin leads to a more occluded or partially buried spin-

label environment[5], as would be found at inter- or intra-tau interfaces. However, there is no significant decrease in water exposure as probed by the nitroxide moiety tethered to the peptide, as judged by the magnitude of the ESEEM effect. This indicates that the spin labels may experience weak tertiary contacts, but do not become entirely buried. Note that soft pulses of 40 and 80 ns lengths were used to suppress local spin concentration effects to the echo decays[6]. This observation agrees well with the expectations based on the results from fluorescence spectroscopy of ThT-active fibrils (Fig. S3).

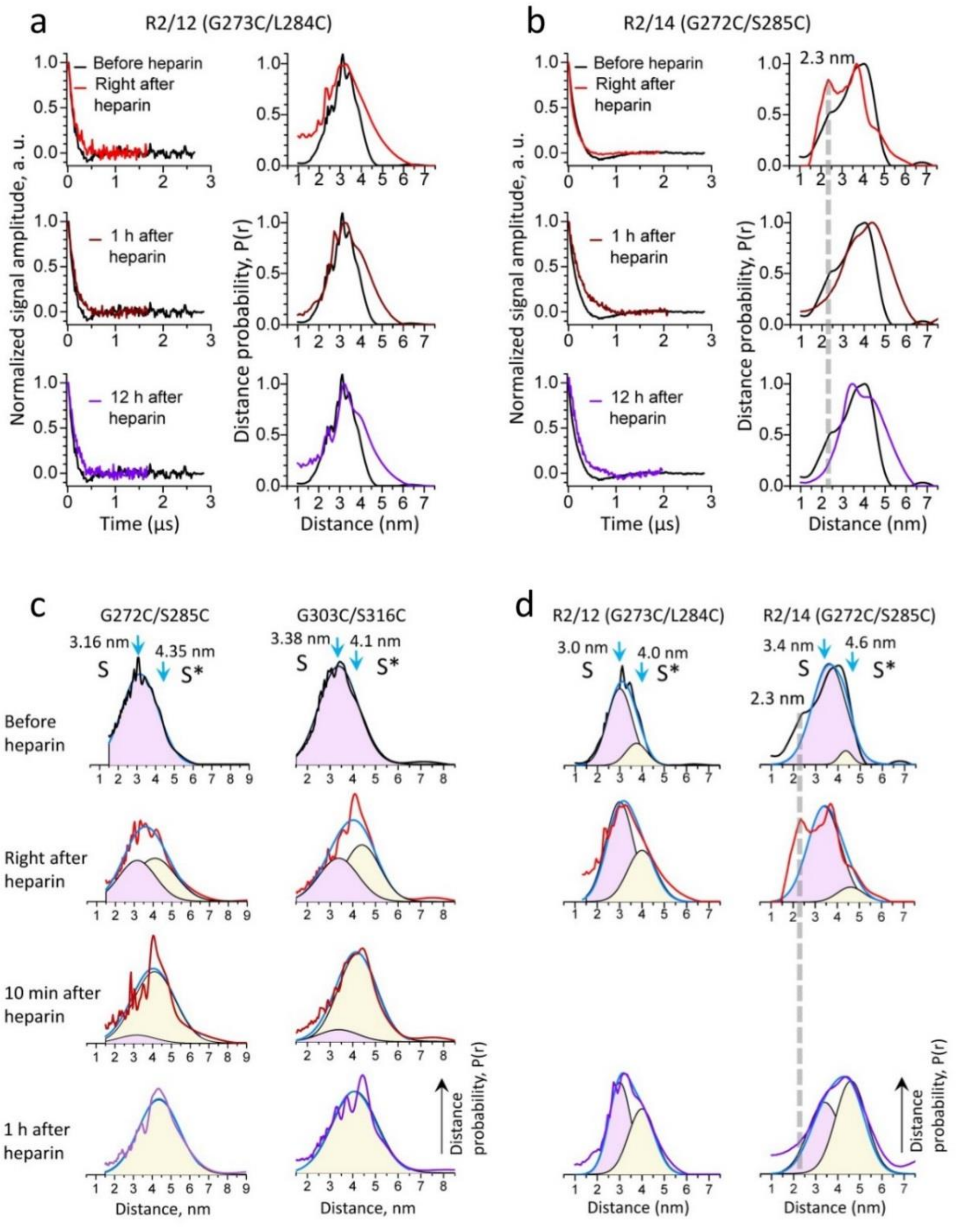

Supplementary Figure S6. DEER time-domain data and distance distributions for peptide samples and Gaussian fits of the distributions for all analyzed samples. (a, b) Background subtracted time-domain DEER data (respective left panels) and reconstructed distance distributions (right panels) of spin-labeled R2/12 G273C/L284C (in a) and R2/14 G272C/S285C (in b). In each panel, the data obtained in the absence of heparin (black) are overlaid with the data obtained after incubation with heparin (red, brown, and purple) with incubation times as indicated. The DEER signals are normalized to unity at zero time and the distance distributions are normalized to unity at maxima. (c, d) The experimental distance distributions fit to either one or two Gaussians for DEER data of Δtau187 variants (in c) and tau peptides (in d). The experimental DEER-derived distances are shown for spin-labeled tau constructs G272C/S285C and G303C/S316C and for R2 tau peptides, G273C/L284C (denoted R2/12) and G272C/S285C (denoted R2/14), before and after ~1 min (i.e. "right after"), 10 min, and 1 h of incubation following the addition of heparin. The respective P(r)'s colors are black (before heparin), reddish (1 and 10 min), and purple (1 h); the envelopes of Gaussian fittings are shown in blue, while the individual Gaussians corresponding to S and S* conformations are filled with lilac (S) or beige (S*). The transition from more compact to extended conformation upon incubation with heparin is thus made more obvious by using Gaussians, although the process is clearly seen just by looking at the progress of *P*(r)'s. The small, but visible, peaks at 2.3 nm in P(r) marked with the dashed line are due to a well-known electron-deuterium ESEEM effect caused by deuterated solvents in the sample, which induces oscillations at 4 MHz (shown in Fig. S5) at Ku band (17 GHz), and thus should be ignored. For a point of reference, the same electron-deuterium ESEEM effect leads to oscillations at 2 MHz at X band, corresponding to 3 nm in the P(r) distribution.

The experimental distances in Fig. S6 were fitted to either one- or two-Gaussian models using the nonlinear list squares curve fitting algorithm in Origin software package (OriginLab). The Gaussian fittings are plotted in blue together with the experimental data in the colors noted above. The Gaussian means characterizing S and S* states are indicated by blue arrows and numbers in the figure for Δtau187 and tau peptides. The Gaussian widths and standard deviations are compiled in Table 1 (presented in the main text). Based on the increase in the content of long distances, manifested as a build-up at the long-distance edge of the distributions, the estimates from Gaussians fittings of DEER distance data strongly support the S-to-S* transition model for both Δtau187 and tau peptides. The Gaussian envelopes present better matches for the DEER data

of Δtau187 mutants than the DEER data for tau peptides, whose $P(r)$'s are more complex, particularly for R2/14 peptides. The use of a harder, 16 ns pump pulse, led to a visibly narrow artifact peak at 2.3 nm superimposed to the DEER-derived P(r) distribution (see Fig. S5). This is a well-known and well-understood artifact peak caused by electron-deuterium nuclear hyperfine couplings leading to electron spin echo envelope modulation (ESEEM) at the ^{2}H NMR frequency of 4 MHz at there here employed Ku band (17 GHz), corresponding to 2.3 nm in P(r). This artifact is present in all DEER experiments (except at very high fields where the forbidden transitions leading to ESEEM effects are suppressed), as long as deuterated solvents are employed, and is more or less apparent, depending on the exact experimental condition, data reconstruction techniques and overlap with the P(r) distribution. Both peptides showed the presence of population at shorter distances (the band in 2-3 nm range), in line with the expectations from MD simulations; but this consideration makes the Gaussian fit a less-than-ideal representation of $P(r)$'s of the fitted components. Nevertheless, the reconstructed time-domain signals indicate that this is a minor issue (see Fig. S7). Thus in our fittings these short distances were disregarded. The exact nature of these minor effects may require future study, but such study is outside the context, focus and scope of this work.

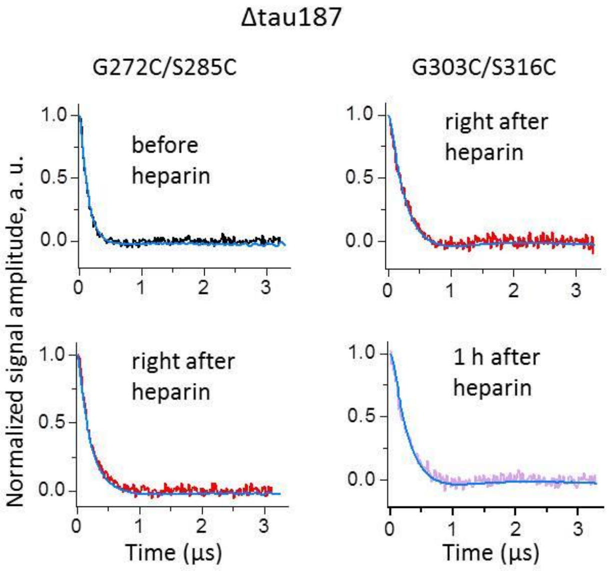

Supplementary Figure S7. The time-domain DEER data, computed using $P(r)$'s based on two Gaussian fittings (in blue), are overlapped with the experimental time-domain DEER data for representative samples of Δtau187 G272C/S285C and G303C/S316C variants. In all cases, the

experimental and calculated DEER data from the fit show excellent agreement. The results validate the hypothesis that the representation of P(r) as a model based on two Gaussians accurately reconstruct the experimental time-domain data. The peptide data yield very similar results as do the proteins, and thus we only display selected and representative data.

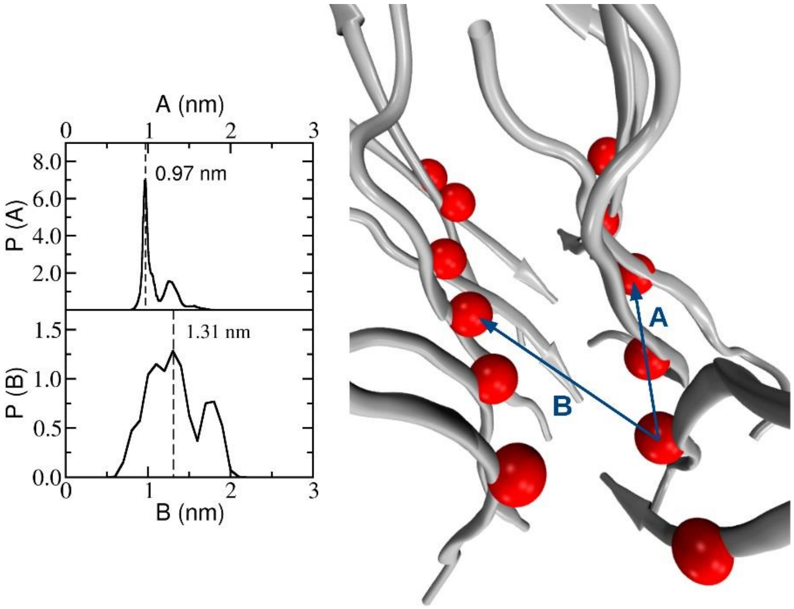

Supplementary Figure S8. Right: Top view of a 2-layer fibril formed by anti-parallel beta-sheets of anti-parallel dimers of R2/WT peptide. Snapshot taken at t=20 ns of a 100 ns long MD simulation. Alpha-carbons of the ASN-279 residues are highlighted in red. The next-to-next neighbor distances (within a layer) and the next-cross neighbor distances (across the layers) between the alpha-carbons of the ASN-279 residues are denoted by A and B respectively. The corresponding distributions of A and B are shown on the left. The most probable distances for A and B are 0.97 nm and 1.31 nm respectively.

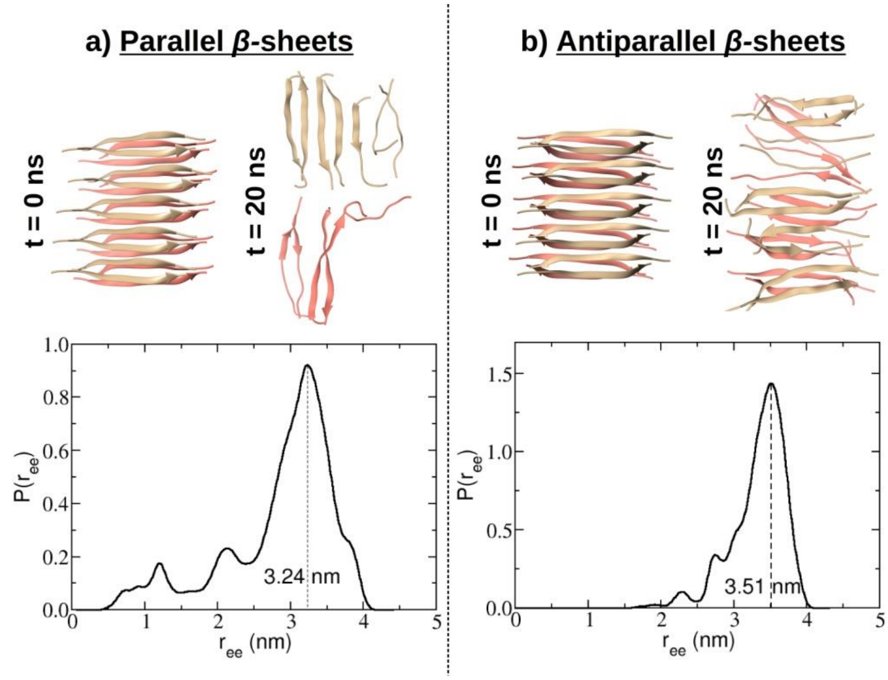

Supplementary Figure S9. Shown are the end-to-end distance distributions of the peptide chains in fibrils formed by two a) parallel and b) antiparallel β-sheets, consisting of anti-parallel dimers of R2/WT peptides using MD simulation. Fibrils were simulated for 100 ns. The snapshots are shown at 0 ns and at 20 ns, as the parallel construct is no longer stable after 20 ns. Fibril consisting of antiparallel β-sheets was found to be more stable, maintaining fibrous structures throughout the entire 100 ns simulation. The most probable end-to-end distances of the peptides for the parallel and the antiparallel β-sheets were found to be 3.24 nm and 3.51 nm respectively. Peptides in the parallel β-sheets were less extended on average as the fibril consisting of the parallel β-sheets was less stable and the data reflect a relatively high population of the smaller oligomers.

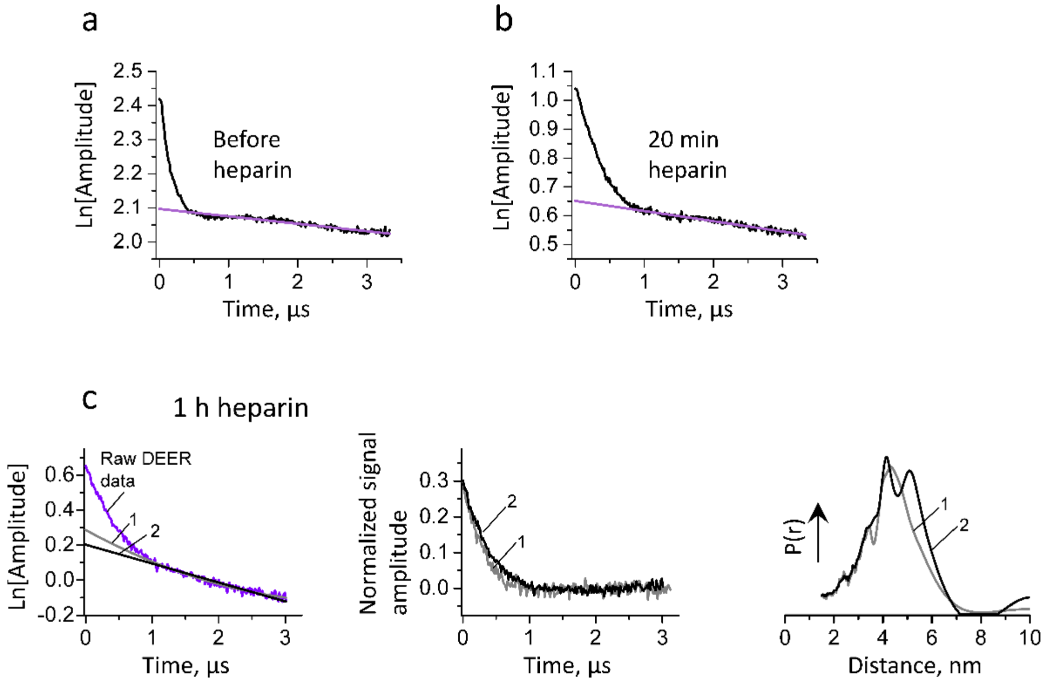

Supplementary Figure 10. The extent of variations of reconstructed *P*(*r*) due to uncertainties in background subtraction of the time-domain DEER data is illustrated with a case study of processing the raw 17.3 GHz DEER data of the G272C/S285C mutant of Δtau187. (a, b) Time-domain data for the samples without heparin and at 20 min after the incubation with heparin. Linear fits to the baseline (purple) are shown and appear as a good match to the background. (c) The 1 hour after heparin addition data is analyzed in greater detail because the baseline slope is a factor of 4 greater compared to that in (b), likely due to increase in tau fibril population. For any sample that contains even a small tau fibril population that need to be accounted for (i.e. heparin-treated samples) a 2nd order polynomial may be considered for the baseline for which a stretched exponential is expected. In the left most plot, we thus compared the fits to the baseline using either the 2nd order polynomial (baseline 1 in gray) or a linear fit (baseline 2 in black). The linear background (baseline 2) subtraction results in a small increase in the modulation depth (center panel) and broader distance distribution (right most panel). Notably, the polynomial fit (baseline 1) after applying the L-curve Tikhonov method does not produce negative excursions[7,8] in the P(r) distribution at long distances, in contrast to what is observed after a linear fit (baseline 2), suggesting that long-range couplings in fibrils do contribute to non-linear baselines. Hence, the 2nd degree polynomial is a better option to fit the baseline of the time-domain DEER data from tau after heparin addition. However, the difference between these two baseline correction methods

is still minor, partly due to a sufficiently long recording time of >3 μs, reducing the uncertainty. We thus reconstructed the distance distribution, P(r), of all heparin-treated samples of Δtau187 (as shown in Fig. S6) by applying a 2nd order polynomial background fit to their baselines to avoid the destabilizing effect of unwanted intermolecular contributions. For all Δtau187 samples without heparin and all peptide samples, P(r) was reconstructed after subtracting a linear baseline on a semi-log scale from the time-domain DEER data.

Supplementary Materials and Methods

Thiovlavin T (ThT) Fluorescence

ThT fluorescence was measured using a Tecan M220 Infinite Pro plate reader using an excitation wavelength of 450 nm and an emission wavelength of 488 nm. Samples were prepared by mixing 25 μM tau with 6.25 μM heparin supplemented with 6.7 μM ThT.

Continuous-wave EPR and lineshape analysis

CW-EPR measurements were completed on Δtau187 labeled at site 322C with MTSL at X-band (9.8 GHz) using a Bruker EMX spectrometer with attached dielectric cavity (ER3123D). A 4 μL sample comprised of 800 μM tau with 200 μM heparin was loaded into a quartz capillary (Vitrocom) and placed inside the EPR cavity. EPR spectra were recorded while using ~6 mW of microwave power, 0.3 G of modulation amplitude and a sweep width of 150 G.

CW-EPR data were simulated using the MultiComponent software of Dr. Christian Altenbach (University of California, Los Angeles). In each spectrum, the **A** tensors were set to $A_{xx} = 6.2$, $A_{yy} = 5.9$ and $A_{zz} = 36.4$ while the **g** values were fixed to $g_{xx} = 2.0078$, $g_{yy} = 2.0058$, and $g_{zz} = 2.0022$. The rotational correlation time of the mobile component was determined from EPR before addition of heparin to the sample. The Heisenberg spin exchange frequency (ω) was allowed to vary in order to obtain a single-line component. Simulation of each EPR spectrum was then completed by fitting the following parameters: rotational correlation time of the immobile and β-sheet components, order parameter S and the relative populations of each of the three components.

Supplementary References

1 Goedert, M. *et al.* Assembly of microtubule-associated protein tau into alzheimer-like filaments induced by sulphated glycosaminoglycans. *Nature* **383**, 550-553 (1996).

2 Pavlova, A. *et al.* Protein structural and surface water rearrangement constitute major events in the earliest aggregation stages of tau. *Proc. Natl. Acad. Sci. U.S.A.* **113**, E127-136 (2016).

3 Eschmann, N. A. *et al.* Tau aggregation propensity engrained in its solution state. *J. Phys. Chem. B* **119**, 14421-14432 (2015).

4 Margittai, M. & Langen, R. Template-assisted filament growth by parallel stacking of tau. *Proc. Natl. Acad. Sci. U.S.A.* **101**, 10278-10283 (2004).

5 Huber, M. *et al.* Phase memory relaxation times of spin labels in human carbonic anhydrase ii: Pulsed epr to determine spin label location. *Biophys. Chem.* **94**, 245-256 (2001).

6 Sato, H. *et al.* Impact of electron-electron spin interaction on electron spin relaxation of nitroxide diradicals and tetraradical in glassy solvents between 10 and 300 k. *J. Phys. Chem. B* **112**, 2818-2828 (2008).

7 Chiang, Y. W., Borbat, P. P. & Freed, J. H. The determination of pair distance distributions by pulsed esr using tikhonov regularization. *J. Magn. Reson.* **172**, 279-295 (2005).

8 Chiang, Y. W., Borbat, P. P. & Freed, J. H. Maximum entropy: A complement to tikhonov regularization for determination of pair distance distributions by pulsed esr. *J. Magn. Reson.* **177**, 184-196 (2005).
